# Behind Tolerance and the Cost of Coexistence: The Cd–Mn Interaction in Sunflower Triggers Translocation Reconfiguration, Impacting Productivity and Food Security

**DOI:** 10.1021/acs.jafc.5c14272

**Published:** 2026-07-24

**Authors:** Aline Aparecida Silva Pereira, Mateus Moreira Bernardes, Victor Navarro Silva, Rafael Agostinho Ferreira, Fernanda Carlota Nery, Eduardo Gusmão Pereira, Elisa Monteze Bicalho

**Affiliations:** † 67739 Universidade Federal de Lavras/UFLA, Depto. de Biologia, C.P.3037, Lavras, Minas Gerais 37200-900, Brasil; ‡ 74383 Universidade Federal de São João Del Rei/UFSJ, Depto. de Biotecnologia, São João Del Rei, Minas Gerais 36301-160, Brasil; § Universidade Federal de Viçosa/UFV, Instituto de Ciências Biológicas e da Saúde, Campus Florestal, Florestal, Minas Gerais 35690-000, Brasil

**Keywords:** trace elements, bioaccumulation, toxicity, food chain, PTE-related risks

## Abstract

Soil bioavailability influences plant uptake of chemical elements, but accumulation and translocation vary among species, particularly in tolerant plants such as sunflower. Because essential and potentially toxic trace elements persist in soils and enter the food chain, this study investigated the Cd–Mn interaction beyond apparent tolerance, evaluating its effects on metal translocation, photosynthetic efficiency, productivity, and food safety. Chlorophyll fluorescence, gas exchange, conversion efficiency, dry matter, oil content, pigments, and Cd and Mn concentrations in soil, roots, leaves, and seeds were evaluated. Mn uptake decreased in the presence of Cd, whereas Cd uptake increased with Mn. Mn accumulated mainly in leaves, while Cd remained in roots but showed greater translocation to shoots with Mn. Despite preserved photosynthetic function, high Mn reduced seed dry matter, conversion efficiency, and oil content, with smaller reductions under combined Cd exposure. Cd translocation to seeds raises food safety and biomagnification concerns.

## Graphical abstract

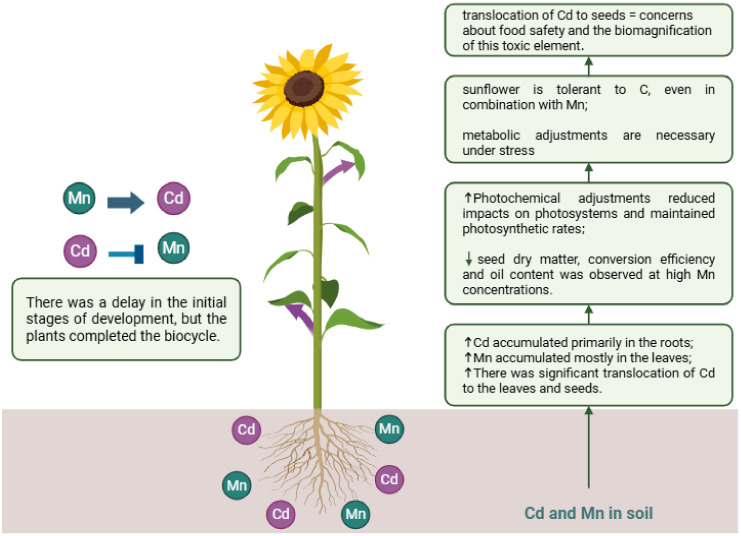

## 1 Introduction

Soil contamination by trace elements is described as one of the biggest environmental concerns of the last decades.^1^ In soils, chemical elements occur naturally,^2,3^ and most of the trace elements (TEs) are also important nutrients for higher plants in various stages of metabolism, such as in gene regulation,^4^ cell signaling, CO_2_ assimilation, stomatal conductance, and synthesis of specialized metabolites.^5^ However, the intensification of anthropogenic activities such as mining, excessive phosphate fertilizer use, and untreated effluent discharge has contributed to an increase in their concentration, making them potentially harmful to plant species and the food chain.^6,7^

In addition to the occurrence of chemical elements in the soil, multiple elements’ simultaneous combination, concentration, and bioavailability of ions to the roots interfere with the potential for absorption and accumulation by plants.^8^ However, the persistent nature of nonessential and potentially toxic elements can generate risks and harm to animals and plants at different levels.^9^ A large portion of polluted soils is used for agricultural production,^10^ and as a result, the exposure of plants to soils contaminated with trace elements has consequently increased, which poses risks to the biota and food chain.^11,12^ Therefore, factors such as agricultural sustainability, productivity, and crop production can be considered unsafe due to the persistence and accumulation of potentially toxic elements (PTEs) in soil.^13^

Regarding toxic and nonessential elements for plants in agricultural soils, high concentrations of cadmium (Cd) have become a serious public health problem due to its presence and bioavailability for absorption by plants.^14^ Cadmium has a long half-life and is highly toxic to living organisms.^12^ It is considered a phytotoxic element even at low concentrations and can be easily transported from the soil to plants and, subsequently, to animals and humans.^15^ The phytotoxicity of Cd induces osmotic stress in plants, resulting in increased production of reactive oxygen species, culminating in damage to membranes and organelles,^16^ in addition to reducing the absorption and transport of essential elements.^17^ Because it is highly mobile, nonbiodegradable, persistent, and mostly bioavailable for absorption, Cd can accumulate in plant tissues and pose an imminent risk to ecosystems,^18,19^ where its accumulation and transfer in the food chain have gained considerable attention in recent years.^20^

It has been discussed^20–22^ that Cd absorption by roots, entry into the xylem, and then translocation to the shoot is mediated by transporters. However, it is known that there are no specific transport channels for Cd in plants and that this and other nonessential elements, such as lead, are commonly transported to plant tissues in competition with essential metal ion transporters, such as those for manganese (Mn), zinc (Zn), and iron (Fe). Transporters such as OsNRAMP1, which is located in the plasma membrane, cooperate in the absorption and accumulation of Mn and Cd in rice, where, due to the cationic similarity of Mn with Cd, its overexpression increases Cd accumulation in this crop.^23,24^

Manganese is a transition metal and an essential micronutrient for plants, playing important roles in plant metabolism, with unique properties, contributing directly and indirectly to plant development.^25^ Through redox state changes, Mn contributes to vital processes such as the electron transport chain and photosynthesis, in addition to being a structural component catalyzing enzymatic reactions.^26,27^ However, this similarity between this essential micronutrient and Cd, which is potentially toxic, can generate competition and interference in several physiological functions.^27^ Through the binding of these metals to biomolecules, biological functions can be impaired or interrupted in addition to preventing or delaying ion absorption and transport processes.^25^

Plant species perceive, emit signals, and tolerate different concentrations of chemical elements in their tissues. Over time, as adaptation traits, some plant species have developed high tolerance to Cd, being able to accumulate concentrations above 100 μg g^–1^ dry weight (DW), which is considered the threshold value for Cd hyperaccumulators.^28^ These species, described as hyperaccumulators, have Cd tolerance and detoxification mechanisms with less or no toxicity effects.^29^ Species classified as hyperaccumulators exhibit tolerance to Cd and efficient detoxification mechanisms, allowing them to accumulate this element with reduced or absent toxic effects. However, even under these conditions, responses to this unfavorable environment can occur, such as alterations in development and reduced yield. Furthermore, when accumulation occurs in organs of commercial interest, it can compromise economic value, either through decreased productivity or devaluation of the product in the market.^30^

Studies have reported remarkable tolerance and potential for accumulating Cd in oilseed species.^31^ Among the oilseed species that are tolerant and effective in removing cadmium from soil, sunflower (*Helianthus annuus* L.) has been described as having high potential.^32^ Sunflower stands out among the oilseeds due to its high oil content in its seeds, with estimated amounts higher than 50%, with about 68% of these being linoleic acid, which is ideal for biodiesel production.^33^ Seeds are also sources of proteins used in the production of rations, fertilizers, and forages.^34^ In this context, trace elements from the soil can be absorbed by plants and translocated to organs of commercial interest, such as oilseeds, where they are partitioned into bran and oil, with greater retention in the bran.^35^ However, when grown in contaminated soils, the economic benefits become limited. Many trace elements in oilseeds remain in the bran produced after oil extraction, where a small fraction is transferred to the oil.^35–37^ This same author also described that Cd enters the food chain through direct consumption of plants or through meat from animals that feed on contaminated sources.

Environmental contamination generally occurs in a complex manner, involving multiple elements; therefore, assessing the absorption and accumulation of a single element in isolation does not adequately represent the actual conditions.^2^ The synergistic composition of soil elements triggers adaptive plant strategies, particularly decreasing the absorption of toxins and lessening physiological damage.^38^ Little is known about how high soil concentrations of Cd, particularly in combination with Mn, affect Cd and essential-microelement uptake, induce physiological changes via absorption-balance disruptions, activate defense mechanisms, mediate shoot translocation, and ultimately influence sunflower productivity and food security. Thus, it was hypothesized that the coexistence of Cd and Mn induces metabolic and physiological adjustments in sunflower that affect the dynamics of absorption and partitioning of elements, with possible repercussions on the productive efficiency and the quality of the final product. In this way, this study aimed to holistically investigate Cd–Mn interaction in sunflower beyond its apparent tolerance, revealing the cost of coexistence by examining how this interaction reconfigures metal translocation from roots to grains, alters photosynthetic efficiency, affects productivity, and impacts food security.

## 2 Materials and Methods

### 2.1 Plant Material and Experimental Conditions

The experiment was carried out in the Plant Physiology Sector of the Universidade Federal de Lavras (UFLA) greenhouse in Lavras/MG, Brazil. Sunflower hybrid seeds (HELIO 250) were used as the plant material.^39^ After being collected, the seeds were stored in multilayer Kraft paper bags and polyethylene plastic bags and in a cold chamber with a constant temperature of 10 °C and water concentration of 9–10%.

Red oxisol with a clayey texture and sand were used in a 2:1 proportion. The substrate was homogenized and placed in 18 L pots. The contamination of the homogenized soil was based on prevention and investigation values for Cd and Mn established by the National Environmental Council (CONAMA) 420/2009 (CdCl_2_),^40^ using cadmium chloride ((CdCl_2_) and manganese sulfate (MnSO_4_) solutions. After soil contamination, the pots were incubated for 21 days. The soil fertilization was according to recommendations of,^41^ and pH was adjusted to between 5.5 and 6.5. Seeds were sown, and the experiment was conducted in a greenhouse for 130 days with an average temperature of 25 °C and relative humidity (RH) of 60%.

The experiment was set in randomized blocks, with 6 treatments: (T1) control, (T2) 1.3 mg kg^–1^ Cd, (T3) 5 mg kg^–1^ Cd, (T4) 400 mg kg^–1^ Mn, (T5) 1.3 mg kg^–1^ Cd + 400 mg kg^–1^ Mn, and (T6) 5 mg kg^–1^ Cd + 400 mg kg^–1^ Mn. The experiment included four replicates for each treatment in each sample carried out.

### 2.2 Analysis of Cd and Mn Concentrations

At the experiment's conclusion, the metal content was measured in soil samples and in accumulated root, leaf, and seed tissues, with four replicates per treatment. Plants were collected and sectioned into root and leaf and washed in running water, in acid solution (0.1% HCl), and again in running water for drying in an air circulation oven at 60 °C for 48 h. Seeds were collected from the inflorescence and kept in an oven at 60 °C until constant weight is reached. After drying, samples were crushed and digested in concentrated nitric–perchloric acid, and later, the Cd, Mn, and phosphorus (P) concentrations were quantified by the methodology described in^42^ using the Atomic Absorption Spectrophotometer.

### 2.3 Evaluation of Bioconcentration and Translocation

Metal bioaccumulation and translocation were calculated to evaluate the bioaccumulation potential and translocation efficiency of Cd and Mn by sunflower plants. Bioconcentration factor (BCF) indicates the plant’s efficiency to absorb and concentrate an element extracted from the soil to accumulate in its tissues.^43^ It is calculated by the following equation:^44^

$$Bioconcentrationfactor\left(BCF\right)=\frac{Metalconcentration\in theplant}{Metalconcentration\in soil}$$

The translocation factor (TF) indicates plant efficiency in translocating the analyzed element accumulated in the roots to the aerial part. The TF was calculated using the following equation^45^ and is presented as a percentage.

$$Translocationfactor(TF)=\frac{Caerial}{Croot}x100$$

### 2.4 Irradiation Measurement

Irradiance was determined through the curve of incident solar energy integral per m^2^ as a function of time and is expressed in W m^–2^ or MJ m^–2^.^46^ A pyranometer (Max. 3000 W/m^2^, Li-Cor Li-200SZ) was used to obtain the global radiation data. The equipment recorded the incident global radiation and the reflected radiation by means of quantometers installed above and below the third fully expanded leaf every 1 h during the entire period of the experiment. At the end, after integrating the data obtained over time, the total incident radiation was calculated using the equation:

$$A=\int_{a}^{b}f\left(x\right)dx$$

where *f*(*x*) determines the area under the curve in the Cartesian plane and *a* and *b* represent the evaluated time interval.

### 2.5 Gas Exchange Analysis

Gas exchange measurements were performed between 8 and 10 h, in the middle third of the third fully expanded leaf of each plant, using infrared gas analyzer model LI-6400XT, LI-COR, Lincoln, NE, USA. Measurements took place under a CO_2_-controlled system (6400-01, Li-Cor Inc.) at 400 μmol mol^–1^, at a leaf temperature of 25 °C and with artificial light adjusted to 1,500 μmol photons m^–2^ s^–1^ that was provided in a 2 cm^2^ area by a light-emitting diode (LED) source (model 6400-02B Red-Blue, Li-Cor Inc.). The following variables were analyzed: net photosynthetic rate (*A*, lmol m^–2^ s^–1^), stomatal conductance (g_s_, mol m^–2^s^–1^), transpiration (E, mmol m^–2^s^–1^), and the ratio between internal and external CO_2_ concentration (Ci/Ca).

### 2.6 Chlorophyll *a* Fluorescence

Chlorophyll *a* fluorescence and photosynthetic pigments were performed throughout the plant life cycle, in vegetative and reproductive period: V4 (4 fully expanded leaves), V8 stage (8 fully expanded leaves), R4 (beginning of inflorescence opening, preceding anthesis), and R7 (onset of seed development). A portable pulse amplitude modulated (PAM) fluorometer (Mini-PAM, Heinz Walz GmbH, Effeltrich, Germany) was used together with a leaf clip holder. Initial fluorescence (F0) and maximum quantum yield of photosystem II (PSII, Fv/Fm) were determined in leaves acclimated to the dark for a period of 30 min.^47^ After the analyses were carried out in the dark, leaves were exposed to a flow of photosynthetic photons of 1000 μmol m^–2^ s^–1^ for 60 s, followed by a saturation pulse to determine the effective quantum yield of PSII (ϕPSII),^45^ photochemical quenching coefficient (qL),^48^ and nonphotochemical quenching of fluorescence (NPQ).^49^ To calculate the apparent electron transport rate (ETR), the following equation was used: ETR = 0.5 × lA × ϕPSII × PPFD, where 0.5 is the assumed proportion of absorbed quanta used by PSII reaction centers^50^ and lA is leaf absorbance.

### 2.7 Biomass Analysis

At the end of the experiment, the accumulated total dry matter, including root, leaf, and seed, was measured.^51^ Samples were placed in Kraft paper bags and taken to a forced circulation oven at 65 °C until a constant weight was reached. Dry mass values were obtained by weighing the plant material on digital scales.

### 2.8 Conversion Efficiency

The conversion efficiency parameter was evaluated based on the equation:

$$Yp=\varepsilon_{i}\times \varepsilon_{c}\times \varepsilon_{p}\times \sum Q$$

*Yp* is the potential crop yield, *ε_i_* is the interception efficiency of leaves, *ε_c_* is the conversion efficiency of radiation to biomass, *ε_p_* is the partition efficiency, and ΣQ is the sum of incident radiation in the evaluation period.

*Yp* was assessed on the basis of the mass of the seeds collected in relation to the total accumulated dry matter.

Interception efficiency (*ε_i_*) was calculated based on the absorbance of the radiation using the equation:

$$\varepsilon_{i}=Q-Qr$$

which *Q* represents the incident radiation and *Qr* the reflected radiation.

Partition efficiency (*ε_p_*) was obtained by the equation as follows:

$$\varepsilon_{p}=MS_{g}/MS_{total}$$

where *MS_g_* is the grain dry matter and *MS*_*total*_ is the total plant dry matter.

For conversion efficiency (*ε*_*c*_), the expected value for sunflower cultivation is 2.46 g MJ^–1^, based on the culture cycle stipulated in the work reported in ref 52. Thus, the factor analysis was performed in the cultivation conditions of the experiment and compared to this reference value.

### 2.9 Evaluation of Achenes Oil Content

Ethereal extract determination was performed according to ref 53 with modifications using a TE-044 Fat Determiner Soxhlet extractor and using petroleum ether as solvent. Sunflower seeds grown in the 6 treatments were used for the analysis. Initially, seed teguments were removed and then the samples were macerated and dried in a circulating-air oven at 105 °C for 1 h. After drying, 1 g of material was weighed into filter paper cartridges. The cartridges were placed inside reboilers plus 100 mL of petroleum ether. Samples remained immersed in the solvent for 3 h at 70 °C. After immersion extraction, the samples were left suspended for 30 min to allow the condensed solvent to drip off. At the end, the cartridges containing the samples were removed and kept in an oven at 70 °C for 1 h for complete evaporation of the solvent. Subsequently, the cartridges were transferred to a desiccator for 30 min and weighed for ethereal extract quantification using the following equation:

EE%=(Initial⁡weight−final⁡weight)×100

### 2.10 Statistical Analysis

Statistical analysis was performed in Rbio software.^54^ Data were submitted to Analysis of Variance (ANOVA), and, in case of normal distribution, Tukey’s test of means was performed at a 5% significance level. Data that did not meet the normality assumptions were analyzed using generalized linear models (GLM), without the need to assume a normal distribution. Subsequently, the means were compared using Tukey’s test at a 5% significance level. Principal component analysis (PCA) was performed using tidy verse, stats, and fact extra R packages.

## 3 Results

In the presence of Mn, there was a greater accumulation of Cd in roots, leaves, and seeds (Figure 1A–C). For Mn, the highest concentration was found in leaves; nevertheless, the high concentrations in the leaves and seeds stand out (Figure 1B). In the treatment with 400 mg of Mn alone, there was a greater accumulation. When combined with Cd, Mn concentrations decreased—particularly in the seeds, where they were approximately 80% lower. The highest accumulation of Cd was observed in roots from plants in all treatments, when compared to leaves and seeds (Figure 1D,E,F). Being a potentially toxic element, Cd showed a high level of accumulation in the roots. As can be seen, in the presence of Mn, there was a greater absorption and bioaccumulation of Cd in all plant organs and concentrations analyzed.

**Figure 1:**
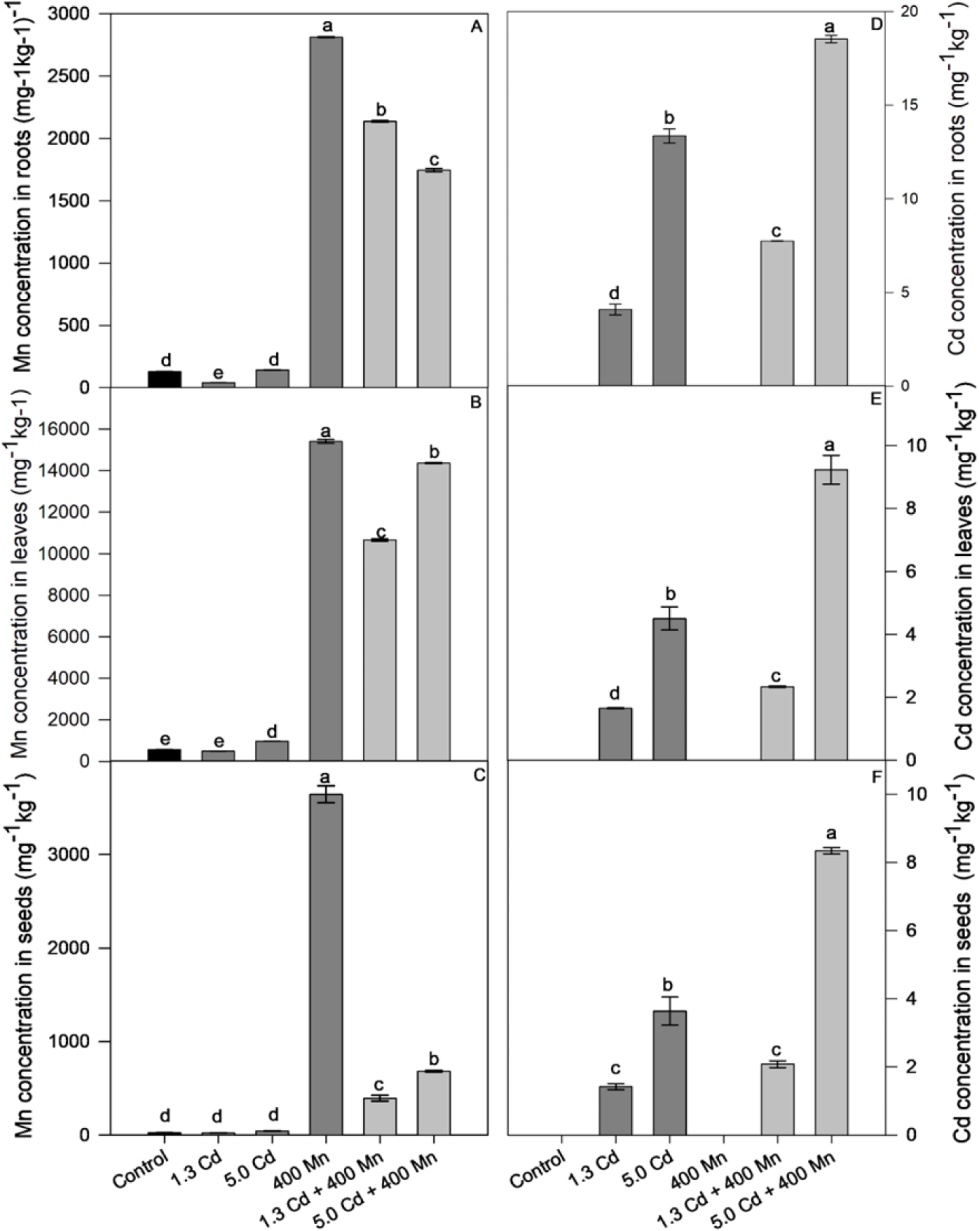
Mn concentration in roots (A), leaves (B), and seeds (C) and Cd in roots (D), leaves (E), and seeds (F) of sunflower (*Helianthus annuus* L.) cultivated in different treatments with a Cd concentration of 1.3 or 5.0 mg kg^–1^, Mn concentration of 400 mg kg^–1^, and their combinations. Values are means ± standard error (*n* = 4). Equal letters demonstrate that there was no statistical difference between treatments by the Tukey test (*p* < 0.05).

It was observed that, in the presence of Cd and in isolation, there was a greater bioaccumulation of Mn (Figure 1B). However, when simultaneously introduced from the soil, Cd negatively interferes with the absorption and bioaccumulation of Mn. When observing the translocation from the root to the shoot, it was found that there is good Mn transfer when this element occurs in conjunction with a higher concentration of Cd (Figure 1A). Interestingly, the treatment containing only Mn showed less bioaccumulation and transfer to the aerial tissues compared to the treatment containing only Cd.

When observing the bioaccumulation and transfer factors for Cd, the responses were opposite to those found for Mn. Cadmium bioconcentration factors were significantly higher in the presence of excess Mn, by at least 59% compared to Cd alone (Figure 2D). The treatment containing the highest concentration of Cd in conjunction with Mn, and both concentrations where there is only excess Cd, showed the highest translocation rates to the aerial tissues (Figure 2C). It can be noted that the highest absorption rate does not necessarily reflect the highest translocation rate for cadmium (Cd). In the presence of Mn, Cd was more absorbed and bioaccumulated, but at the concentration of 5 mg·kg^–1^ of Cd associated with Mn was the highest translocation rate to the aerial part.

**Figure 2:**
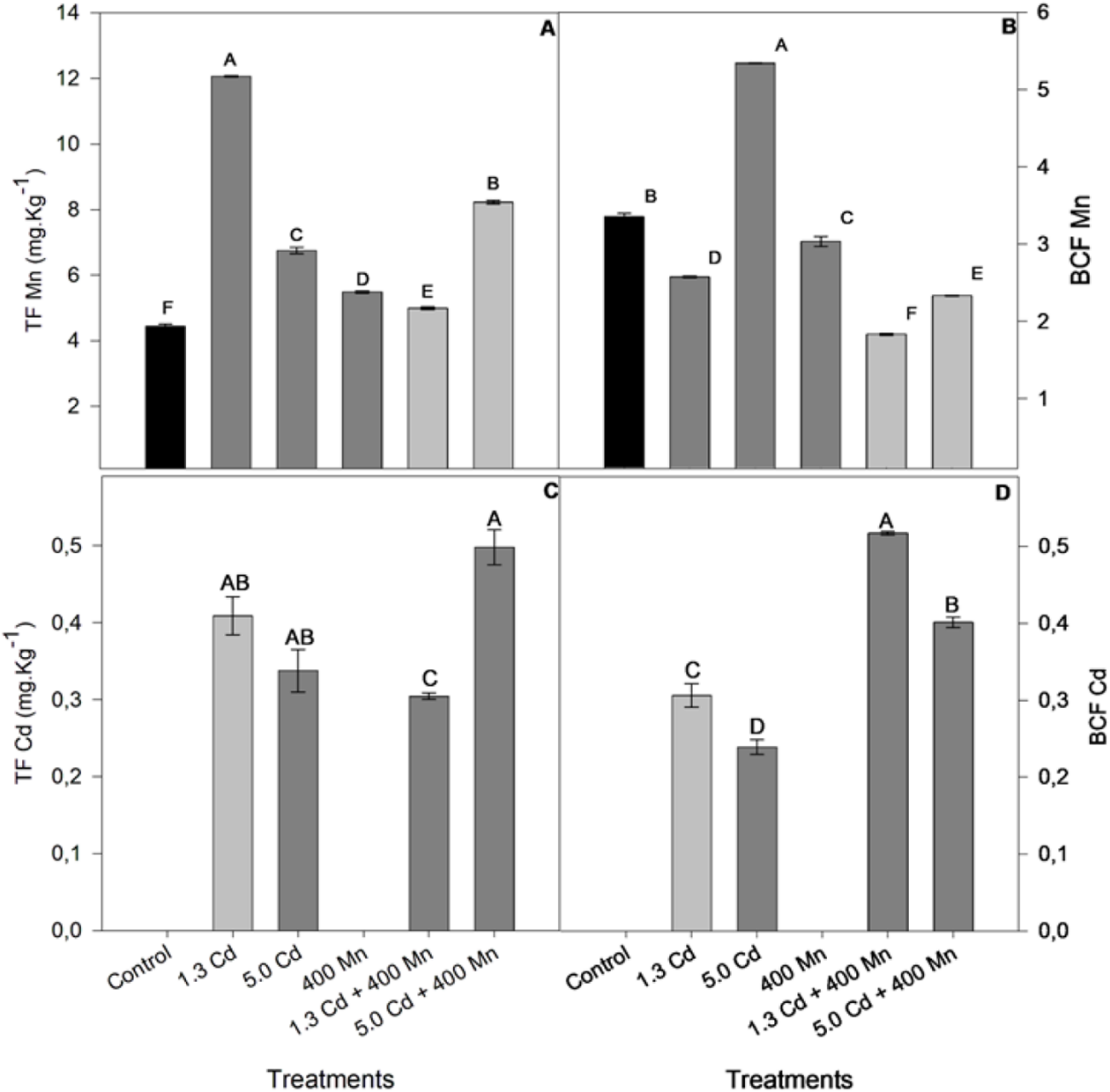
Mn bioconcentration factor (A), Mn translocation factor (B), Cd bioconcentration factor (C), Cd translocation factor (D), P translocation factor (E), P bioconcentration factor (F), and tolerance index (G) of sunflower (*Helianthus annuus* L.) plants grown in Cd- and Mn-contaminated soil. Equal letters show that there was no statistical difference between treatments by Tukey’s test (*p* < 0.05). Bars represent means ± standard error (*n* = 4).

Different responses were observed regarding gas exchange in the cultivation conditions and stages of development analyzed. The CO_2_ assimilatory rate (*A*) (Figure 3A) and stomatal conductance were lower in plants exposed to Mn during all periods analyzed. The treatment containing 1.3Cd + 400Mn presented high rates of *CiCa*, in comparison to the other treatments; however, it presented a reduced rate of *A*. The stomata conductance remained high until the end of the vegetative stage (V8) with a reduction in the reproductive rate as well as the transpiration rate.

**Figure 3:**
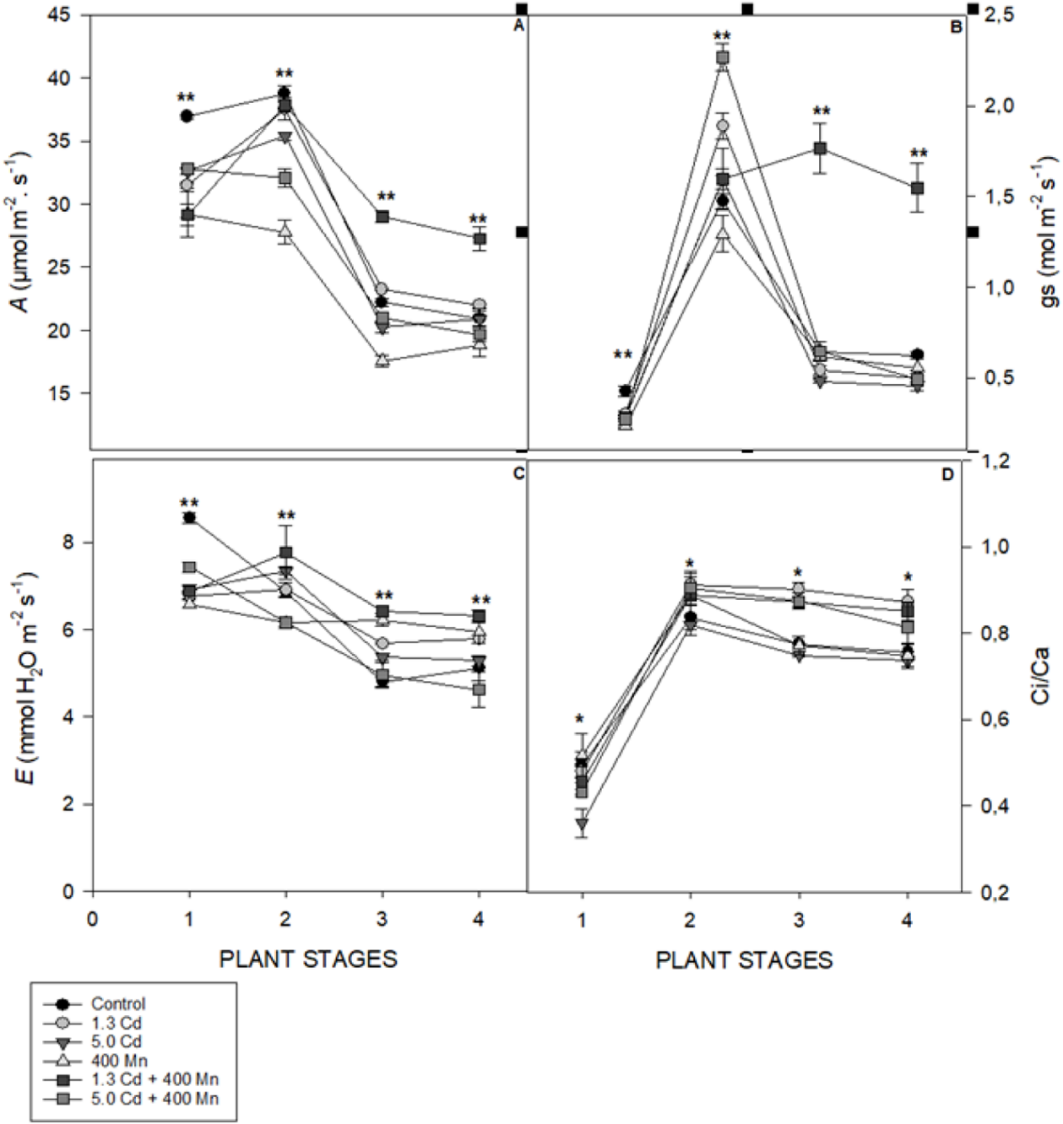
Photosynthesis (A), stomatal conductance (B), transpiration (C), and internal and atmospheric carbon ratio (D) in sunflower (*Helianthus annuus* L.) leaves grown in soil contaminated by Cd and Mn. Shading in gray indicates analysis performed during the reproductive period. One asterisk indicates a significant difference in relation to the stage of development and interaction with the time factor by Tukey’s test (*p* < 0.05). Bars represent means ± standard error (*n* = 4). On the *x* axis: 1 = V4; 2 = V8; 3 = R4; 4 = R7.

Interestingly, the treatment with 5 Cd + 400 Mn showed high rates of *A*, *CiCa*, and *gs*. As observed in the other treatments—including the control—assimilation (*A*) and transpiration (*E*) rates were higher during the vegetative stages than in the reproductive period. However, only the 400 Mn treatment maintained elevated transpiration rates over time. The highest *gs* values were observed at the V8 stage, followed by a decrease, except for the 1.3 Cd + 400 Mn treatment.

The lowest ratio of internal and external CO_2_ concentrations (Ci/Ca) (Figure 3D) for all treatments was observed at the V8 developmental stage, close to the transition between the vegetative and reproductive periods. During the reproductive stage, only the 5.0 Cd treatment showed a significant reduction of Ci/Ca. No significant differences were observed between the treatments at each developmental stage. The alterations and adjustments were correlated only with the stage of the plants, without these alterations being caused by the treatments.

The maximum quantum yield of PSII (Fv/Fm) did not show significant differences between treatments in each measurement (Figure 4A). The electron transport rate (ETR) (Figure 4B) and the effective quantum yield of PSII (ϕPSII) (Figure 4C) showed interaction between time and treatments. There was a significant increase over time in both until the R4 developmental stage, which was later reduced in the R7 stage. The treatment 1.3 mg Cd + 400 mg Mn presented the lowest ETR in the initial stage of development, having later becoming equal to the other treatments and remaining until the R7 phase. The highest ETR and ϕPSII were identified in stages V8 and R4, the transition period between the vegetative and reproductive phases. Regarding nonphotochemical quenching (NPQ) (Figure 4D), all treatments exhibited similar temporal patterns, with values peaking at the final vegetative stage (V8) compared to the initial stages. However, the treatment with 400 mg of Mn stands out. As can be seen, this treatment presented higher NPQ at all stages of development, especially at the end of the vegetative and reproductive stages. These data are consistent with the lower net photosynthetic rate observed.

**Figure 4:**
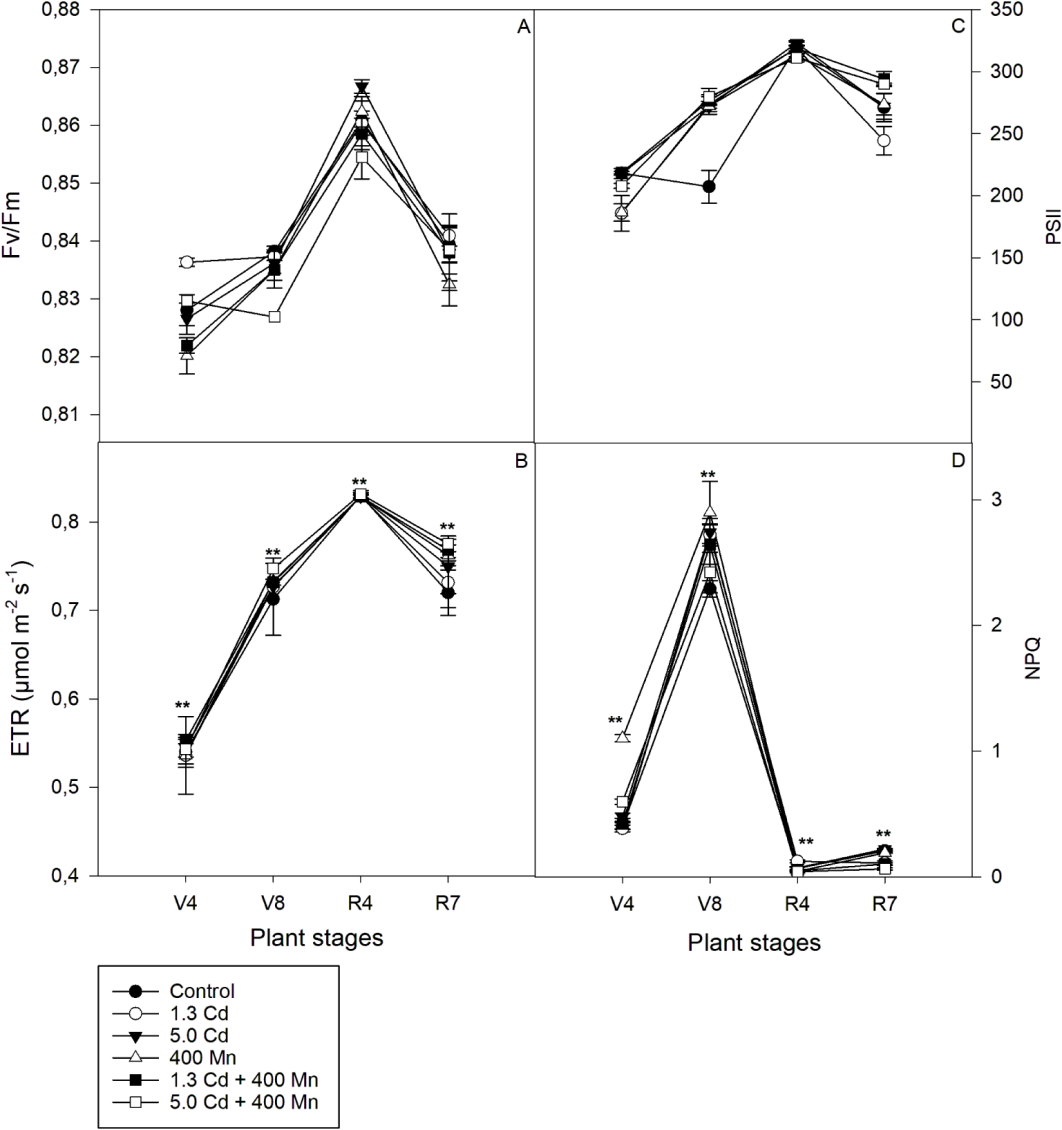
Maximum quantum yield of PSII (Fv*/*Fm) (A), apparent electron transport rate (ETR) (B), effective quantum yield of PSII (C), and nonphotochemical quenching (D). Analyses performed on sunflower leaves (*Helianthus annuus* L.) cultivated in different treatments with a Cd concentration of 1.3 or 5.0 mg kg^–1^, a Mn concentration of 400 mg kg^–1^, and their combinations. Values are means ± standard error (*n* = 4). The gray shading indicates the collections carried out in the reproductive period. One asterisk indicates a significant difference between treatments and two asterisks indicate difference between treatments and interaction with the time factor by Tukey’s test (*p* < 0.05).

Regarding the efficient conversion of photoassimilates into biomass, the tolerance index and gas exchange data were proportionally consistent with accumulated dry weight, root-to-shoot ratio, and achene weight (Figure 5). For the total dry weight parameter (Figure 5), the lowest conversion rate into biomass was found in plants grown in the conditions with 400 mg of Mn in soil and 5.0 mg of Cd + 400 mg of Mn. Sunflower plants grown in soil containing only 1.3 and 5.0 Cd showed results similar to those of the control.

**Figure 5:**
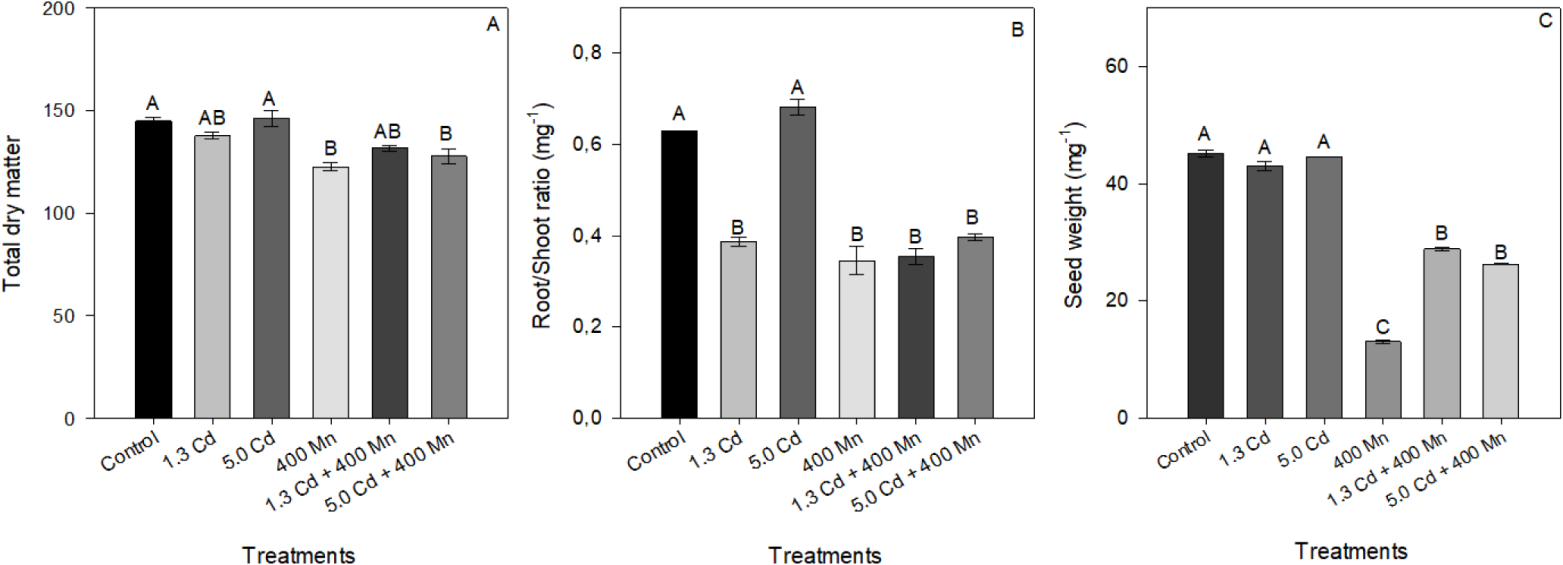
Total dry matter (A), root-to-aboveground ratio (B), and seed weight (C) of sunflower (*Helianthus annuus* L.) grown in Cd- and Mn-contaminated soil. Letters compare the responses in each treatment. Equal letters show that there was no statistical difference between treatments by Tukey’s test (*p* < 0.05). Bars represent means ± standard error (*n* = 4).

The root/shoot ratio was lower in the treatment containing 400 mg of Mn and in the treatment containing 5.0 mg of Cd and 400 mg of Mn together (Figure 5B). The lowest concentration of Cd (1.3 mg of Cd) presented the same value of the ratio when exposed alone and together with Mn. Interestingly, at a concentration of 5.0 mg Cd, the root/shoot ratio was the same as that presented by the control treatment. When the organ of economic interest of the species was observed, the filling of the grains and, consequently, the weight of the achenes were lower when Mn was present (Figure 5C). The treatments containing Cd were similar to the control, but when Cd and Mn were used synergistically, there was a significant reduction.

For the parameter conversion efficiency of carbon fixation by photosynthesis (Figure 6A), there was a statistical difference only in the treatment with 400 mg of Mn. This result reflects what was observed in the parameters of gas exchange and stomatal conductance for this treatment, which were also lower. In addition, when we observed the energy disposition by nonphotochemical quenching, the 400 Mn treatment presented higher values, reflecting that the absorbed energy was being released in other ways than those for carbon fixation and conversion. The treatments containing Cd and Cd added with Mn presented a similar response to the control treatment, not being statistically different. As for the oil content in the achenes (Figure 6B), the control treatment showed the highest values, with the other treatments not differing from each other. However, it is important to emphasize that it was necessary to have a larger number of achenes to reach the weight to quantify the oil in the 400 Mn treatment, reflecting the responses already mentioned and the lower photosynthetic rates and conversion efficiency.

**Figure 6:**
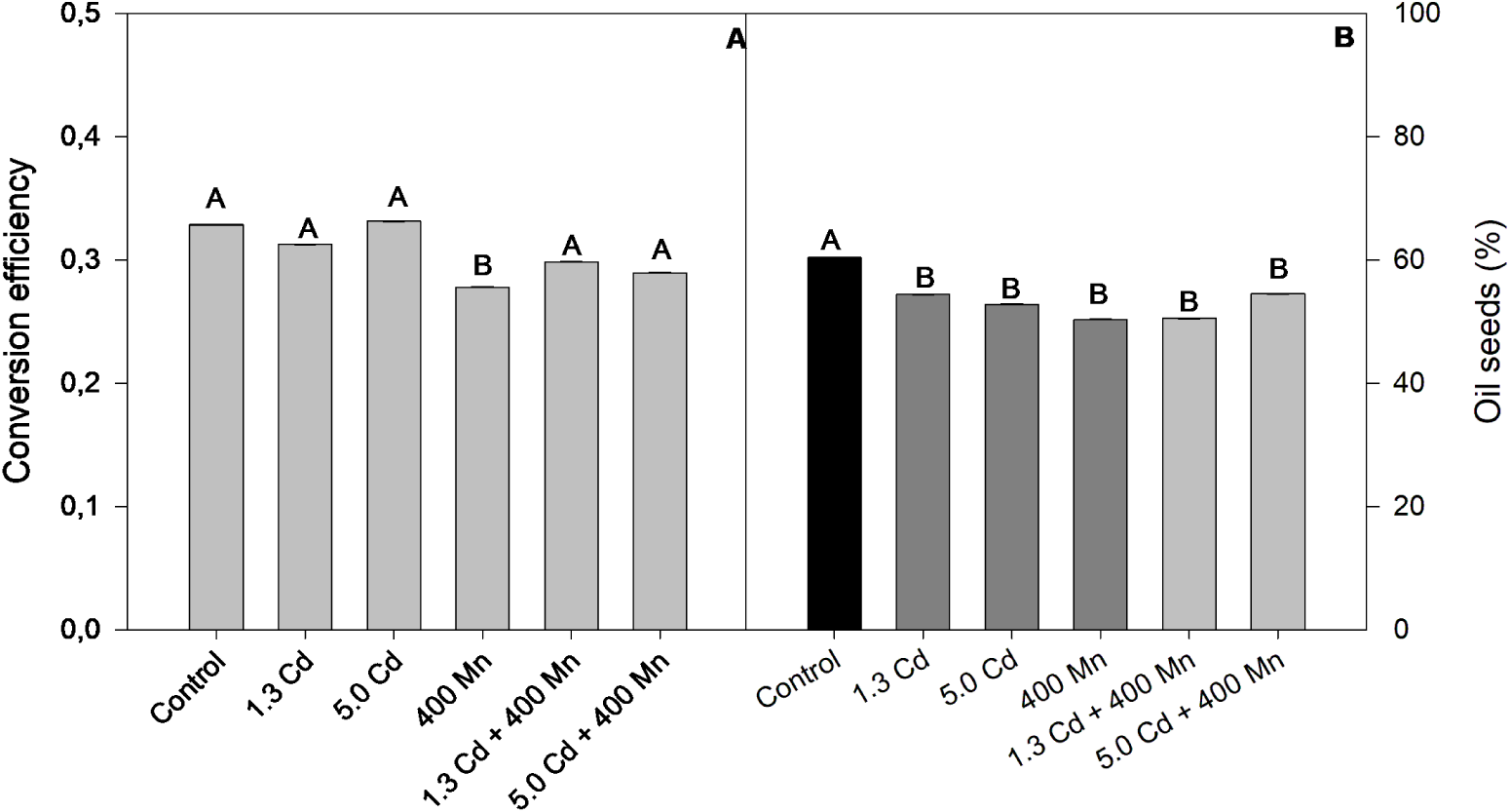
Conversion efficiency (A) and percentage of oil (B) of sunflower (*Helianthus annuus*L.) seeds grown in soil contaminated by Cd and Mn. Letters compare the responses in each treatment. Equal letters show that there was no statistical difference between treatments by Tukey’s test (*p* < 0.05). Bars represent means ± standard error (*n* = 4).

Principal component analysis revealed a clear separation between variables associated with metal dynamics (FT and FBC to Cd) and those related to productive performance, such as seed mass, conversion efficiency, and root-to-shoot ratio, indicating a trade-off between Cd accumulation/translocation and productivity (Figure 7). Combined Cd and Mn treatments shifted toward the translocation axis, corroborating that Mn favors Cd mobility in the plant, consistent with increased translocation to the shoot. On the other hand, high Mn concentrations were negatively associated with productive parameters and oil content, demonstrating greater sensitivity of these attributes to stress.

**Figure 7:**
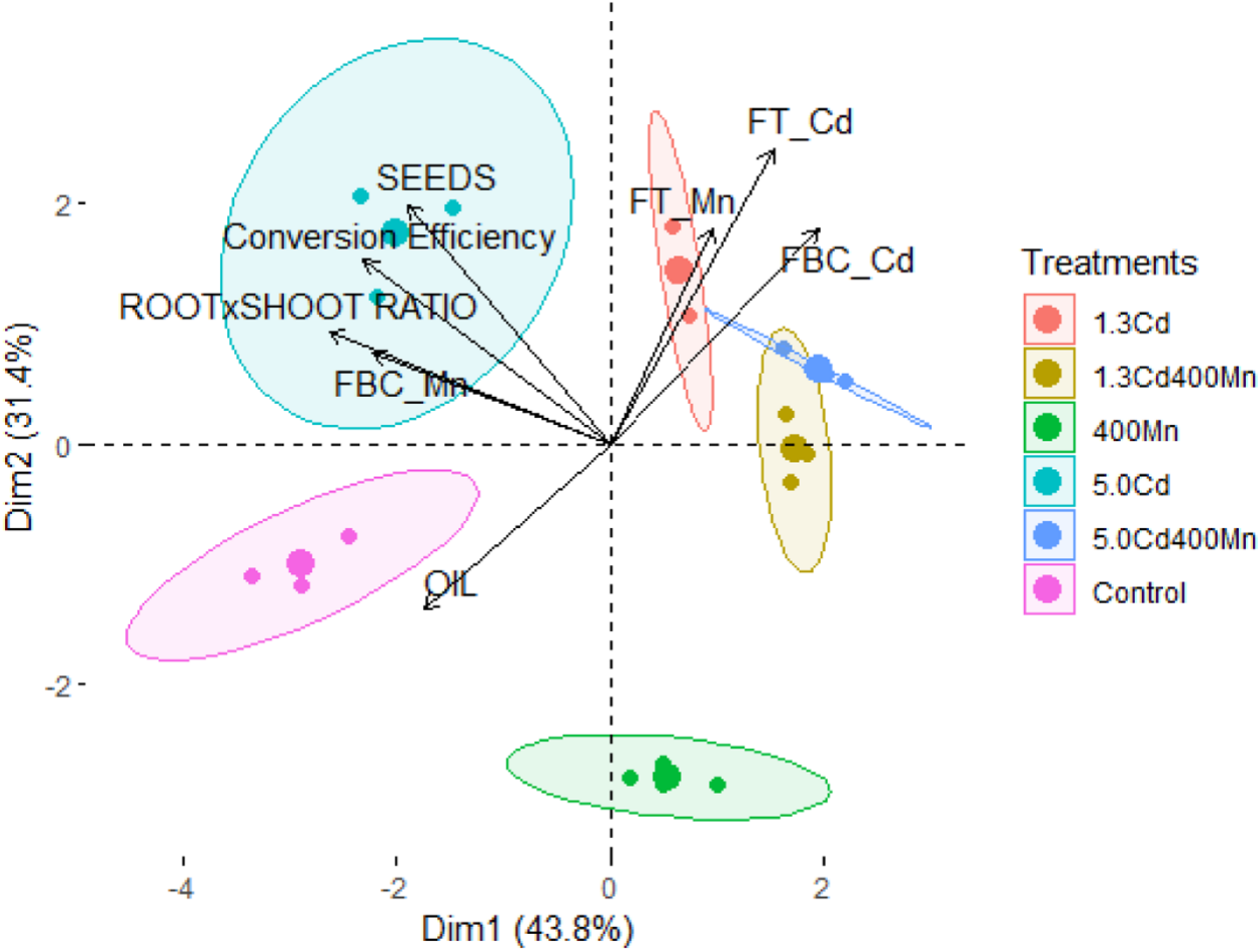
Presentation of the interrelationship between FBC, FT, seed weight, oil content, conversion efficiency, root/shoot ratio, and total dry matter for the six treatments analyzed through Principal Component Analysis.

Despite this, the clustering of the isolated Cd treatment close to physiological variables suggests sunflower tolerance to this metal, without significant impacts on productive performance, reinforcing the absence of a hormetic effect. Taken together, the results indicate that, under multielemental stress, there are metabolic adjustments that prioritize metal transport and compartmentalization mechanisms at the expense of productivity. Additionally, the strong association of Cd with translocation variables highlights the risk of redistribution of this element to organs of agronomic interest such as seeds, implying potential risks to food security.

By monitoring the life cycle of sunflower plants and observing the responses over time, we were able to detect the phenotypic changes that occurred during the developmental stages (Figure 8). It can be inferred that there was a developmental delay in sunflower plants grown in soil containing Mn at the concentration evaluated and under the condition where there was a synergistic availability of Mn and Cd from the initial stages of development. However, under all conditions evaluated, the plants completed the biocycle. This delay can be linked to the defense and response mechanisms presented by the plants in their vegetative and reproductive stages. The maintenance of photosynthetic rates and conversion efficiency even under stressful conditions and the increase in NPQ only at specific developmental stages demonstrate that the observed facts were correlated with the biocycle phase, a direct source–sink relationship. Furthermore, this aspect indicates an investment in biomass and greater photoprotection and defense in plants. These changes were observed especially in the presence of Mn at high concentrations, while Cd presented a hormesis effect.

**Figure 8:**
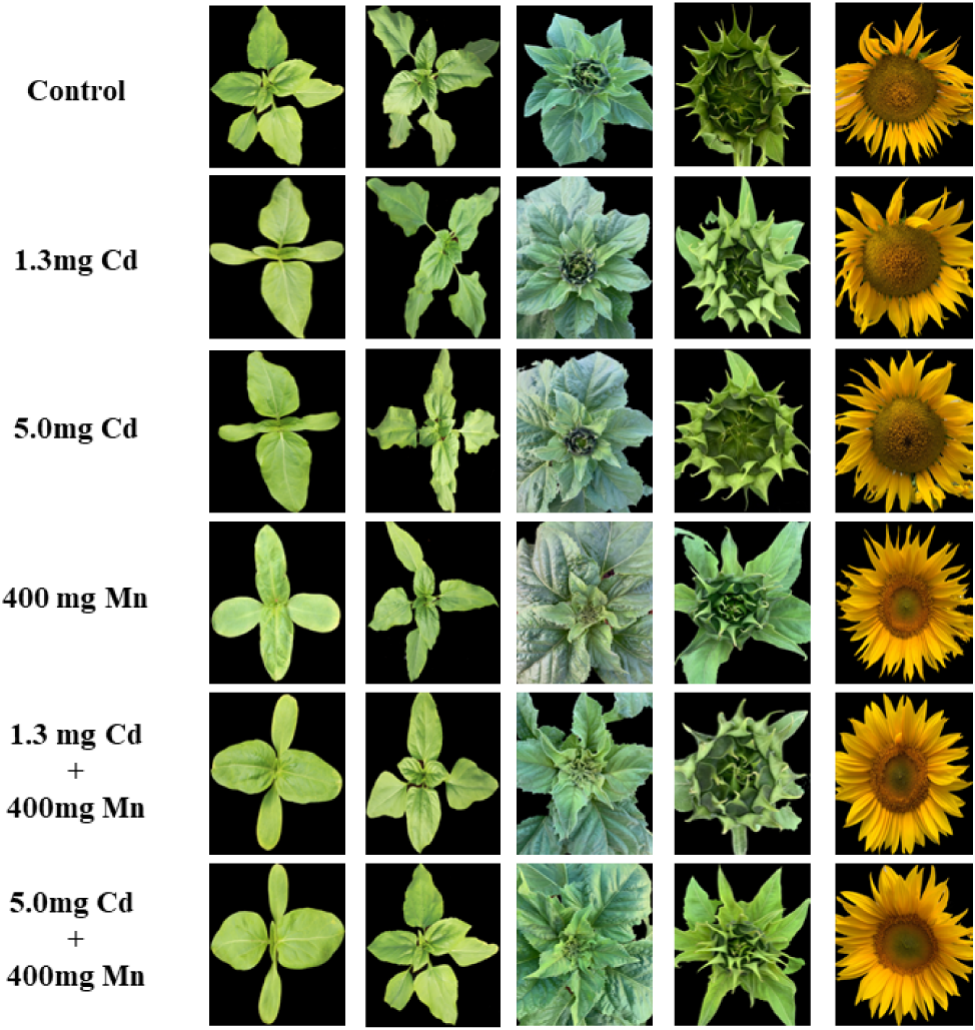
Characterization of the different developmental stages of sunflower (*Helianthus annuus* L.) plants grown in soil with different concentrations of Cd and Mn.

## 4 Discussion

The results found in this study demonstrate that sunflower plants show different strategies to tolerate soil excess Cd and Mn, when arranged individually or synergistically. Curiously, Cd interfered negatively with the uptake of Mn when arranged together. The first strategy of plants when cultivated in areas with high concentrations of potentially toxic elements is the exclusion or complexation of these elements in the roots.^55^ However, sunflower plants adopted the strategy of accumulating Cd and Mn in the tissues of their aerial parts. It is described that Cd is a hitchhiker element, which uses transporters of essential elements, such as the microelements Zn and Mn, in all stages of the pathways of these nutrients, that is, from absorption to the loading of grains or seeds.^56^ The lower uptake of Mn in plants when exposed together with the concentration of 1.3 mg of Cd can be explained by the responses already induced in the root, either in the plasma membrane or by competition between the elements for membrane transport channels, corroborating the results presented regarding absorption and translocation levels for Mn and Cd.^57,58^

In nonstressful concentrations, or in tolerant plants, Cd can induce defense responses in plants.^59^ Moderate Cd-induced stress involves a similar or better performance of plants in the presence of the element when compared to the control and to other potentially toxic elements, a phenomenon called the hormesis effect, as observed in ref 60 in tomato plants. Our results corroborate those provided in ref 60 which allows inferring that Cd tolerance depends on genotype-specific mechanisms, not depending exclusively on the lower concentration in plant tissues. At physiological levels, the hormesis effect induced by Cd is associated with adjustments in photosynthetic activity and in the different forms of energy dissipation, in addition to the change in root length to provide a balance in the nutritional state of plants.^61^ In our results, adjustments were observed in the absorption and translocation rates of the elements under analysis, in the root/shoot ratio, and in plant development. Thus, it is possible to perceive that the mechanisms of response of plants to stress are multiple, varying mainly due to the intensity, exposure time, and space where they occur, being controlled by various signaling pathways.

Plant responses to a combination of stresses do not simply represent the sum of the responses to each stressor.^62^ The conditions in the environment can interact in complex ways where a single stress can induce different responses, and on the other hand, different stresses can result in similar changes^63^ or cause favorable responses. In this way, we observe the physiological adjustments presented by the plants regarding the extraction and absorption of the elements when arranged in an isolated and synergistic way and also regarding the adjustments in photosynthetic metabolism under these conditions. It is possible to affirm that, in the concentrations and conditions evaluated, the phytoremediation potential of sunflower is not affected when Cd and Mn are exposed together at the concentrations evaluated.

The positive connection between the synergistic arrangement of Cd and Mn may be related to specific competition for binding and action sites, culminating in the maintenance of the role played by Mn in maintaining the structure of chloroplasts, plant metabolism, adjustments, and prevention of oxidative damage^64^ and pigment concentration. Thus, it mitigates the damage observed at a high concentration of Mn in isolation. The effects of excess Mn and Cd on carotenoids are only at specific stages of development, close to the transition between the vegetative and reproductive phases, and at the end of development, the concentration may be linked to a greater generation of ROS. This pigment is essential in the process of reducing damage caused by ROS, thus reducing the effects of free radicals.^65^ The maintenance of carotenoid levels in other treatments may be associated with the dissipation of excess energy by plants, favoring the membrane integration^66,67^ and chlorophyll molecules, keeping photosynthetic rates,^68^ that favoured the completion of the plant biocycle, even if more slowly.

Due to the toxicity of Mn at high concentrations^69^ and Cd,^70^ leaf chlorosis can be observed, resulting in a reduction in biomass. As previously reported, Cd at low concentrations can cause leaf chlorosis, tissue damage, and a reduction in biomass.^71^ However, as a tolerance strategy, it is observed that even with a reduction in pigments, the destination of the light energy captured by them was used for the maintenance of gas exchange, requiring less dissipation by chlorophyll fluorescence. Likewise, excess Mn in the tissues can alter metabolic processes, causing oxidative stress and reducing photochemical efficiency, gas exchange, and energy use for plant growth. The concentrations of Cd alone, in this work, did not affect photosynthesis, which was contrary to observations of ref 72. However, 400 mg of Mn, when exposed singularly and together with Cd, caused changes in photosynthesis, especially in the initial stages of development evaluated. This fact can be associated with the reduction of biomass, the root-to-aerial part ratio, and the plants’ achenes cultivated under these conditions. As reported, the results demonstrated that Mn could influence photosynthetic parameters, particularly the destination of electrons and chemical energy from the excitation of chlorophylls. And as explored in ref 73, when not directed to photochemistry, the energized electrons are allocated to oxygen or dissipated, which reduces energy efficiency and conversion.

The biochemical responses induced by high Cd and Mn concentrations can be associated with disruptions in energy metabolism that directly impair plant growth and development.^69,74^ Indeed, phytotoxic levels of these metals have been reported to reduce Rubisco activity and photosystem II efficiency, promote apoplastic accumulation and macromolecular binding in leaf tissues, and constrain stomatal conductance, ultimately limiting CO_2_ uptake.^75^ In this work, the monitoring of changes over time in electron transport and in the quantum yield by plants in the different treatments showed that the conversion of light energy into chemical energy remained efficient, protecting the photosynthetic system, as also reported in ref 23 for wheat.

Species considered as hyperaccumulators, such as sunflower, have a greater capacity for translocation of Cd from the roots to the aerial part, compared to other nonaccumulator species.^29^ Thus, it is understood that plants have different potential for tolerance or sensitivity to Cd, with different cellular defense and detoxification mechanisms being employed, depending on the variety.^30^ In addition to the nonbiodegradable characteristic, the dynamics of essential and nonessential trace elements are controlled by characteristics such as soil properties, pH, cation exchange capacity, and the occurrence and concentration of the elements present therein,^76,77^ as in the present study. Changes in natural conditions can enable the biomagnification of these elements,^6^ thus, toxicity and bioavailability for the trophic chain can be affected by environmental physical–chemical characteristics and interactions between the elements present there.^78^

In addition to the tolerance potential of cultivars, another crucial point concerns the development stage at which plants are subjected to stress and the responses presented by individuals throughout the biocycle.^79^ The work carried out in ref 80 is in line with the results observed for the tolerant sunflower species, where, under prolonged Cd stress, plants still show growth retardation and substantial loss in final crop yield. The phytotoxic potential of Cd, which culminates in impaired plant growth, is largely associated with the reduction of photosynthetic pigments and cell elongation,^81^ with biomass being considered a direct indicator of plant tolerance to this element.^30^ On the other hand, despite being an essential element and having a specific function in photosynthesis, Mn in isolation increased the NPQ and consequently the reduction in the photosynthetic rate and the efficiency of photochemical conversion, which significantly reduced the biomass in the roots, leaves, and seeds of plants grown under these conditions. This indicates that, although Cd has no defined function, it was less phytotoxic than Mn at the concentrations evaluated, and sunflower plants showed a hermetic effect at the concentrations evaluated. This fact is altered when Cd and Mn are added synergistically to the soil.

A trend that has been growing over the past few years has focused on the search for economic benefits in the use of edible crops that are also hyperaccumulators and tolerant to high concentrations of trace elements, aiming at a sustainable production strategy integrated with the recovery of degraded agricultural areas.^82,83^ Among the species, oilseeds demonstrate a high potential for tolerance and capacity to accumulate Cd.^84^ However, our results demonstrated alarming data regarding food safety and the potential use of byproducts, such as seeds for oil extraction and bran production. All this is due to the half-life characteristics, nonbiodegradable nature of Cd in the parts of commercial and edible interest, and subsequent biomagnification, in the food chain, which can be a threat to agricultural production.^85^ In addition, the need to promote food security is the third Sustainable Development Goal (SDG) adopted by the United Nations General Assembly for the year 2030.
